# Modulation of Specific Sensory Cortical Areas by Segregated Basal Forebrain Cholinergic Neurons Demonstrated by Neuronal Tracing and Optogenetic Stimulation in Mice

**DOI:** 10.3389/fncir.2016.00028

**Published:** 2016-04-20

**Authors:** Irene Chaves-Coira, Natali Barros-Zulaica, Margarita Rodrigo-Angulo, Ángel Núñez

**Affiliations:** Departamento de Anatomía, Histología y Neurociencia, Facultad de Medicina, Universidad Autónoma de MadridMadrid, Spain

**Keywords:** diagonal band of Broca, nucleus basalis magnocellularis, cholinergic projections, cholinergic facilitation, cortical evoked potentials, transgenic mice

## Abstract

Neocortical cholinergic activity plays a fundamental role in sensory processing and cognitive functions. Previous results have suggested a refined anatomical and functional topographical organization of basal forebrain (BF) projections that may control cortical sensory processing in a specific manner. We have used retrograde anatomical procedures to demonstrate the existence of specific neuronal groups in the BF involved in the control of specific sensory cortices. Fluoro-Gold (FlGo) and Fast Blue (FB) fluorescent retrograde tracers were deposited into the primary somatosensory (S1) and primary auditory (A1) cortices in mice. Our results revealed that the BF is a heterogeneous area in which neurons projecting to different cortical areas are segregated into different neuronal groups. Most of the neurons located in the horizontal limb of the diagonal band of Broca (HDB) projected to the S1 cortex, indicating that this area is specialized in the sensory processing of tactile stimuli. However, the nucleus basalis magnocellularis (B) nucleus shows a similar number of cells projecting to the S1 as to the A1 cortices. In addition, we analyzed the cholinergic effects on the S1 and A1 cortical sensory responses by optogenetic stimulation of the BF neurons in urethane-anesthetized transgenic mice. We used transgenic mice expressing the light-activated cation channel, channelrhodopsin-2, tagged with a fluorescent protein (ChR2-YFP) under the control of the choline-acetyl transferase promoter (ChAT). Cortical evoked potentials were induced by whisker deflections or by auditory clicks. According to the anatomical results, optogenetic HDB stimulation induced more extensive facilitation of tactile evoked potentials in S1 than auditory evoked potentials in A1, while optogenetic stimulation of the B nucleus facilitated either tactile or auditory evoked potentials equally. Consequently, our results suggest that cholinergic projections to the cortex are organized into segregated pools of neurons that may modulate specific cortical areas.

## Introduction

Acetylcholine (ACh) is essential to normal central nervous system (CNS) function, modulating the activity of the thalamocortical network in many important brain functions, such as arousal (e.g., Buzsáki et al., 1988; Détári, 2000; Szymusiak et al., 2000; Lee et al., 2004; Goard and Dan, 2009), attention (Chiba et al., 1999; Sarter et al., 2003), learning (Wilson and Rolls, 1990a,b; Mayse et al., 2015) and memory (Pauli and O’Reilly, 2008; Hasselmo and Sarter, 2011; Luchicchi et al., 2014; Sarter et al., 2014). Moreover, ACh enhances synaptic plasticity in the hippocampus (Doralp and Leung, 2008; Fernández de Sevilla et al., 2008; Navarrete et al., 2012) and neocortex (Metherate and Ashe, 1993; Kuo et al., 2009; Bueno-Junior et al., 2012; Núñez et al., 2012; Barros-Zulaica et al., 2014; Martin-Cortecero and Núñez, 2014).

In the CNS, ACh transmission is mainly guaranteed by dense innervation of cortical and subcortical regions from disperse groups of cholinergic neurons within the basal forebrain (BF) and the pontine-mesencephalic nuclei. The BF contains a diverse population of neurons, including cortically-projecting cholinergic and noncholinergic neurons as well as various interneurons (Zaborszky et al., 2012). The BF includes the medial septum, horizontal and vertical limbs of the diagonal band of Broca (HDB and VDB, respectively), the substantia innominata (SI), and the nucleus basalis magnocellularis (B), which provide the majority of the cholinergic innervation to the sensory, motor and prefrontal cortices and hippocampus (Semba and Fibiger, 1989; Semba, 2000; Zaborszky et al., 2012, 2015).

Early anatomical descriptions of cholinergic projections were consistent with the notion of a diffuse pathway from the BF to the cortex (Saper, 1987). Nearly all cortical areas and regions are innervated by BF cholinergic neurons (Eckenstein et al., 1988; Lysakowski et al., 1989; Callaway and Henriksen, 1992; Golmayo et al., 2003). However, newer evidence concerning the BF system indicates the existence of a highly structured and topographic organization of efferent projections to sensory cortices (Zaborszky, 2002; Golmayo et al., 2003; Zaborszky et al., 2005, 2015). The above mentioned authors propose that cholinergic and noncholinergic projections to the neocortex are not diffuse but instead are organized into segregated or overlapping neuronal groups (Zaborszky et al., 2015).

Studies measuring cortical ACh level have demonstrated that visual stimulation causes much greater ACh release in visual cortex than in non-visual cortical areas (Collier and Mitchell, 1966; Fournier et al., 2004; Laplante et al., 2005). However, anatomical tracing methods have not revealed any extensive projections from sensory relay nuclei to the BF (Semba et al., 1988; Zaborszky et al., 1991). Thus, it has been proposed that sensory information arrives at the BF through cortico-cortical projections from primary cortical sensory areas via the prefrontal cortex (Zaborszky et al., 1997). Results from both electrophysiological recordings (Golmayo et al., 2003) and inactivation of the prefrontal cortex (Rasmusson et al., 2007) have demonstrated that the prefrontal cortex is necessary for sensory-evoked cortical ACh release. These results strongly support the proposed specific pathway –sensory cortex to prefrontal cortex to BF– for each sensory modality.

In this study, we used retrograde anatomical procedures to demonstrate the existence of specific neuronal groups in the BF involved in the control of specific sensory cortices. Fluoro-Gold (FlGo) and Fast Blue (FB) fluorescent retrograde tracers were deposited into the primary somatosensory (S1) and primary auditory (A1) cortices in mice. In addition, we used an optogenetic method for selective stimulation of cholinergic neurons in the BF of transgenic mice to study the effect of selective stimulation of BF cholinergic neurons on cortical activity. Our studies suggest that cholinergic projections to the cortex are organized into segregated and overlapping pools of neurons that may modulate specific cortical areas.

## Materials and Methods

All animal procedures were approved by the Ethical Committee of the Autonomous University of Madrid, in accordance with European Community Council Directive 2010/63/UE. Efforts were made to minimize animal suffering as well as to reduce the number of animals used. Animals were housed in groups of two to four per cage in a temperature-controlled room with a 14/10 light/dark cycle. Food and water were provided *ad libitum*.

### Anatomical Procedures

The anatomical pathways linking BF with cortical areas were studied by injecting or depositing the neuroanatomical fluorescent retrograde tracers (FlGo; Fluorochromes, Llc. Denver, CO, USA) and (FB; Polysciences, Inc. Warrington, PA, USA) in 18 B6.Cg-Tg (Chat-COP_4_*H_134_R/EYFP, Slc_1_8a_3_)5^Gfng^/J mice. For a better understanding of the characteristics of the cortical afferent connections from BF, FlGo injections were made in the S1 cortex and FB deposits in the A1 cortex of the animals.

The mice were anesthetized with an intraperitoneal injection of a mixture of ketamine (70mg/kg) and xylazine (5 mg/kg) maintained with inhalation anesthetic Isofluorane (0.5%, maintenance doses) and placed in the stereotaxic frame. After appropriate craniotomy, 200 nl of a 4% saline dilution of FlGo was injected in the corresponding cortices of the animals by means of a 10 μl Hamilton syringe at stereotaxic coordinates: for S1 (AP −1.46 mm, L 3 mm, DV 1.5 mm) and A1 (AP −2.46 mm, L 4 mm, DV 2.2 mm), according to the Paxinos and Franklin (2003). Deposits of 0.5–1 mm^2^ pieces of absorbable gelatin “Spongostan” soaked in a 1% saline solution of FB were placed on the appropriate cortex in the animals, for 15 min. Animals were treated with the longer-lasting analgesic buprex (0.075 mg/kg) at the end of the experiment. After a survival period of 1 week, animals were anesthetized with an overdose of the same anesthesia and perfused transcardially with 4% paraformaldehyde in 0.1 M phosphate buffer at pH 7.3 followed by increasing concentrations of sucrose solutions (5%, 10%, and 20%) in the same buffer. Brains were stored in 30% sucrose for at least 5 days for tissue cryopreservation and frozen sectioned on the coronal plane at 40 μm; sections were collected in three consecutive ordered series devoted to Nissl staining, fluorescent visualization and ChAT immunocytochemistry.

Series processed for Nissl staining were used for delimiting structures. Sections containing the cerebral cortex of the fluorescent visualization series were studied under a Nikon Axioskop fluorescent microscope. Sections for ChAT immunostaining were incubated with 1:100 goat anti-ChAT primary antibody (Chemicon AB144P) in a solution containing 20% normal rabbit serum, 5% bovine serum albumin (BSA), and 0.5% Triton X-100 in phosphate-buffered saline (PBS) for 36 h. Incubation in the secondary antibody was carried out with 1:200 biotinylated rabbit anti-goat (Chemicon) in the same solution for 2 h and in Elite ABC kit (Vector Laboratories Inc., Burlingame, CA, USA) for 1.5 h before development with 0.05% 3–3′DAB and 0.003% H_2_O_2_. These sections were studied under an optical/fluorescent microscope, so both ChAT positive neurons and fluorochrome labeled cells could be observed.

For a quantitative study, selected sections were analyzed under a confocal microscope (Leica TCSSP5), using the LASAF Software TileScan tool; samples were analyzed using bio-mapping (maximal projections) under both lin405 mm UV and linAr488 mm using a 10x objective for the quantification of neurons in each channel. Images were a stack of sections in maximal projection, but neurons were counted in each individual layer. Maximal projections of the images were analyzed in two channels (UV and green) and the merged image was also studied. The following procedure was used: (1) For neuronal counting in each channel we selected labeled neurons in each section of the region of interest (ROI). We eliminated the nonspecific background, moved the images to eight bits and then smoothed them with the filter to apply the previous segmentation particle analyzer. In some cases it was necessary to use the ROI from the BG subtraction plugin tool and Watershed tool to separate and count labeled neurons correctly. In cases where the particle analyzer results were entirely satisfactory they were then manually reviewed in the Cell Counter plugin; and (2) for the proportion of double labeled neurons, a manual multipoint tool was used on the merged image and separate channels were also used to corroborate the results. In cases of doubt, possible co-localization of channels was assessed in a merged image combining images of the resulting ROIs in the previous section, in both color channels.

### Electrophysiological Recordings

We have used transgenic B6.Cg-Tg (Chat-COP_4_*H_134_R/EYFP, Slc_1_8a_3_)5^Gfng^/J mice; The Jackson Laboratory) mice expressing the light-activated cation channel, channelrhodopsin-2, tagged with a fluorescent protein (ChR2-YFP) under the control of the choline-acetyl transferase promoter (ChAT). Thus, all cholinergic neurons in CNS express the channelrhodopsin-2 and could be stimulated by blue light and were used for optogenetic stimulation of the BF. Young adult mice (3–6 months old) were anesthetized with urethane (1.5 g/kg, i/p). Depth of anesthesia was sufficient to eliminate pinch withdrawal, palpebral reflex and whisker movement and was assessed periodically during the experiment. Local anesthetic (Lidocaine 1%) was applied to all skin incisions and supplemental doses of urethane were given to maintain areflexia. Animals were placed in a Kopf stereotaxic device (David Kopf Instruments, Tujunga, CA, USA) in which surgical procedures and recordings were performed. The body temperature was maintained at 37°C. An incision was made exposing the skull and small holes were drilled in the bone over the barrel and auditory cortices (AP 1–3 mm, L 5–7 mm, DV 0.2–1 mm, and AP −2.5 mm, L 4 mm, DV 2.5 mm from Bregma, respectively) as well as on the BF, HDB (AP 0.14 mm, L 2 mm, DV 4 mm) and B nucleus (AP −0.7, L 2, DV 4).

Single-unit recordings were performed with tungsten microelectrodes (2–5 MΩ, World Precision Instruments, WPI, Sarasota, FL, USA) and the cortical field potential was recorded through tungsten macroelectrodes (<1 MΩ). Unit recordings in BF were also performed by an optrode (see below). Unit firing was filtered (0.3–3 kHz), amplified via an AC preamplifier (P15, Grass Instruments) and sampled at 10 KHz while field potentials were filtered between 0.3–100 Hz, amplified and sampled at 500 Hz.

Signals were fed into a personal computer with the temporal references of the stimuli for off-line analysis with Spike 2 Software (Cambridge Electronic Design, Cambridge, UK). We used Spike 2 Software for the offline spike sorting. The algorithm first performs crude spike detection by capturing windows around events defined by the voltage crossing of a user-defined threshold. Then, spike sorting is performed with a combination of template matching and a principal component analysis-based cluster cutting.

### Sensory Stimulation

Whisker deflections were performed by brief air puffs using a pneumatic pressure pump (Picospritzer) that delivers an air pulse through a 1 mm inner diameter polyethylene tube (1–2 kg/cm^2^, 20 ms duration, resulting in whisker deflections of ≈15°). To avoid complex responses due to deflections of multiple whiskers, these were trimmed to 5 mm in length, so that reproducible responses were evoked. The experimental protocol consisted of 30 pulses delivered to the principal whisker (whisker that gives the highest spike response) at 0.5 Hz (control period). Whisker stimulation was also applied after blue light stimulation of the BF during 30 min. Auditory click stimulation was performed by application of a brief (1 ms duration) square voltage pulse to Sony earphones. The stimuli were presented at a rate of 0.5 Hz at the level 30 dB. Following the baseline recording, stimulation was also applied after blue light stimulation of the BF during 30 min.

### Optogenetic Stimulation

Optical stimulation of ChR2-expressing neurons was achieved with light-emitting diodes (LED; 473 nm; Thomas Recording, Germany) delivered from an optical fiber (core diameter 120 μm) or through an optrode (microelectrode 1–2 MΩ; core diameter 80 μm + optical fiber; core diameter 120 μm) positioned directly above BF area. The LED was triggered with a square-step voltage command. Stimulation was applied by 20 ms pulse trains of 473 nm light at 5 Hz or by a single long-lasting pulse (200 ms). Illumination intensity was <30 mW/mm^2^ at the BF, which is below the damage threshold of ~100 mW/mm^2^ for blue light (Cardin et al., 2010). The stimulation area was very restricted since total transmitted light power was reduced by 50%, after passing through 100 μm of neuronal tissue, and by 90% at 1 mm (Aravanis et al., 2007).

### Data Analysis

The somatosensory or auditory evoked potentials were calculated every 1 min (30 stimuli). The amplitude of the evoked potential was measured from the baseline to the first negative peak. The mean tactile response was measured from the peristimulus time histogram (PSTH) as the number of spikes evoked in the 0–50 ms time window after the onset of the stimulus divided by the number of stimuli. The power spectrum and wavelet transform were also calculated from cortical field potentials. Field potential periods of 30 s were analyzed by Spike 2 Software, using the fast Fourier transform algorithm to obtain the power spectra. The mean power density was calculated for three different frequency bands: δ-band (0.3–4 Hz), θ-band (4–10 Hz), and β-band (10–30 Hz). Every 30 s the percentage contribution of each band to the global wavelength of the EEG (band power × 100/total band powers) was calculated and normalized against the control value (calculated as the mean value of the 30 s before the blue light stimulation).

Statistical analysis was performed using GraphPad Prism 5 Software (San Diego, CA, USA). Statistical analyses consisted of paired comparisons between the same cells before and after BF optogenetic stimulation. If the data were considered normally distributed, according to the Shapiro–Wilk normality test, we used parametric statistics. For two groups, the *t* test (paired) was used. For multiple comparisons the one-way ANOVA analysis of variance followed by Bonferroni *post hoc* test was used. Data are presented as mean ± standard error of the mean (SEM). The threshold level of significance was set at *P < 0.05 and **P < 0.01 are indicated in figures.

## Results

### Different Neuronal Groups in Basal Forebrain Display Specific Anatomical Pathways to Sensory Cortices

**Figure 1:**
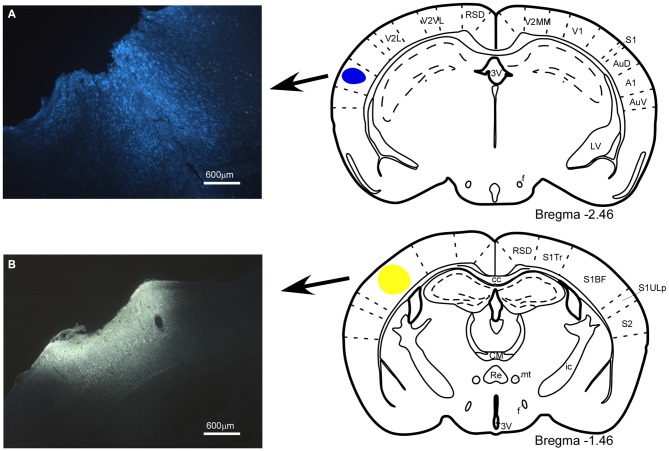
**Location of the injection and deposit of fluorescent retrograde tracers.** Microphotographs of coronal brain sections and schematic drawings showing the injection sites of the retrograde tracers. **(A)** Fast Blue (FB), deposit in the A1 cortex; **(B)** Fluoro-Gold (FlGo), injection in the S1 cortex. In this and in the following figures, abbreviations are: 3V, 3rd ventricle; A1, primary auditory cortex; AuD, secondary auditory cortex, dorsal area; AuV, secondary auditory cortex, ventral area; cc, corpus callosum; CM, central medial thalamic nucleus; f, fornix; ic, internal capsule; LV, lateral ventricle; mt, mamillothalamic tract; Re, reuniens thalamic nucleus; RSD, retrosplenial dysgranular cortex; S1, primary somatosensory cortex; S1BF, primary somatosensory cortex, barrel field; S1Tr, primary somatosensory cortex, trunk region; S1ULp, primary somatosensory cortex, upper lip region; S2, secondary somatosensory cortex; V1, primary visual cortex; V2L, secondary visual cortex, lateral area; V2MM, secondary visual cortex, mediomedial area; V2MM, secondary visual cortex, mediomedial area. Calibration toolbar 600 μm.

**Figure 2:**
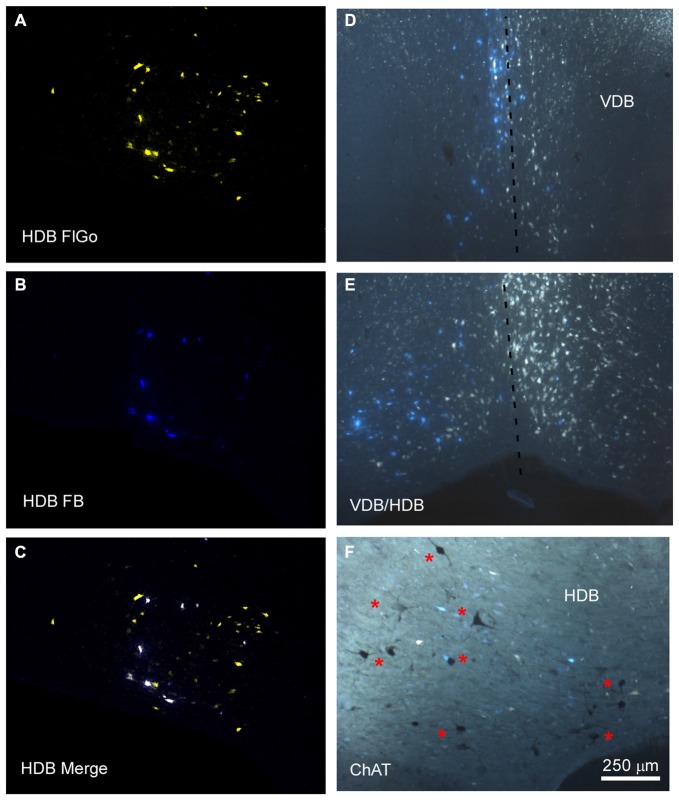
**Distribution of labeled neurons in the VDB/HDB. (A–C)** Confocal microscope images of retrogradely FlGo (S1 injection) and FB (A1 injection) labeled neurons in the VDB/HDB; **(D,E)** fluorescence microscope images of both Fluoro Gold and Fast Blue labeled neurons in the VDB and HDB. Note that some neurons project to both cortical areas. Dashed line indicates the medial hemispheric line. **(F)** Image combining fluorescent microscopy with acetylcholine transferase (ChAT) immunocytochemistry techniques in HDB. Asterisks indicates cholinergic neurons. HDB, nucleus of the horizontal limb of the diagonal band; VDB, nucleus of the vertical limb of the diagonal band. Calibration toolbar for **(A–F)** 250 μm.

**Figure 3:**
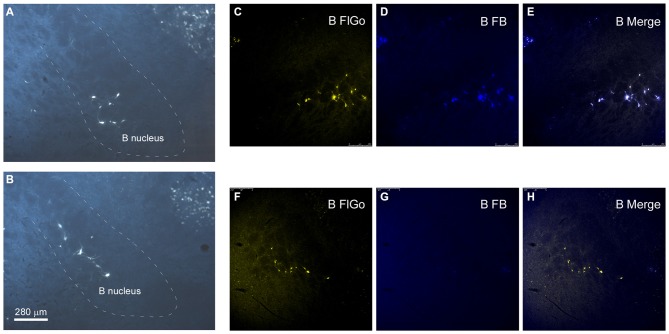
**Distribution of labeled neurons in B nucleus. (A,B)** Microphotographs of coronal sections showing retrogradely-labeled neurons located in B nucleus at two different antero-posterior coordinates. Dashed lines delimitate the B nucleus. **(C–H)** Confocal microscope detail of FlGo (S1 injection) and FB (A1 injection) labeled neurons in B nucleus. Note that some neurons project to both cortical areas. Calibration toolbar for **(A–H)** 280 μm.

The anatomical study of the BF efferent connections to the somatosensory and auditory cortices was performed on 18 cases. The locations of the FlGo injection site in the cortices as well as the FB deposit were confirmed using the sections reserved for fluorescence and Nissl studies (Figure 1). Both injections and deposits were confined to the desired site without signals of diffusion in any case. In all 18 cases the study of the fluorescence series allowed us to detect numerous fluorescent retrogradely single- or double-labeled neurons in the HDB, VDB (Figure 2) or in the SI and B nuclei (Figure 3). The mean number of total labeled neurons in each animal was 1758 ± 234 in VDB/HDB and 56 ± 6 in B nuclei (*n* = 10 mice).

**Figure 4:**
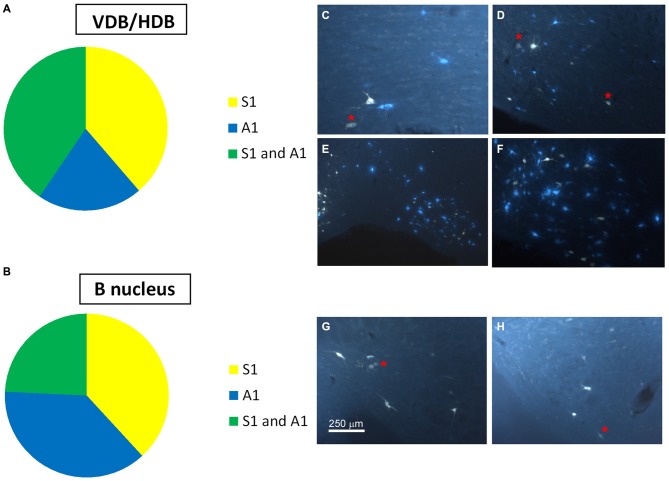
**Distribution of labeled neurons in the VDB/HDB and B nuclei. (A)** Graphic representation of number of labeled neurons in the VDB/HDB after deposit in the S1 and A1 cortices. Note that this BF area mainly project to the S1 cortex. **(B)** Graphic representation of the number of labeled neurons in the B nucleus after deposits in the S1 and A1 cortices. Note that this BF area project to the S1 and A1 cortices in a similar proportion. **(C–F)** Samples of fluorescent microscope images of single- and double-labeled neurons in HDB. **(G,H)** Samples of fluorescent microscope images of single- and double-labeled neurons in SI/B nucleus. Red asterisks indicate double-labeled neurons. Calibration toolbars **(C,D,F–H)** 230 μm, **(E)** 530 μm.

Neurons in the SI and B nuclei were considered together because the two nuclei were difficult to distinguish at caudal levels. Two tracers were deposited in the two different sensory cortices (S1/A1) of the same hemisphere (*n* = 10 mice). This experimental approach allowed us to establish whether the pathway of the BF cortical projecting neurons reaches A1, S1 or both cortices. The microscopy study of VDB/HDB revealed numerous intermingled neurons in these nuclei labeled by FlGo, FB or both tracers (Figures 4C–F). The study of the distribution and percentages of these neurons showed that 34 ± 1.1% of the neurons located in the VDB/HDB were labeled by both fluorescent tracers (neurons projecting to both the S1 and A1 cortices), while most of the VDB/HDB neurons (44.2 ± 7.4%) were single-labeled by FlGo injected into S1 cortex; only 21.8 ± 2.4% of neurons were labeled by FB injected into A1 cortex (Figure 4A). Conversely, the percentage of double-labeled neurons in B nucleus was lower than in VDB/HDB (22 ± 2.1%; Figure 4B), while the percentage of B neurons single-labeled by either one or other tracer was roughly the same (40.6 ± 2.7% from S1 and 37.4 ± 3.1% from A1; Figures 4B,G,H). In addition, the immunochemical study revealed that fluorescent labeled neurons appeared to be scattered among the characteristic cholinergic neurons of the different BF nuclei and some of them were also positive for ChAT immunocytochemistry (Figure 2F).

### Optogenetic Stimulation of Cholinergic Neurons Evokes Different Sensory Cortical Responses

**Figure 5:**
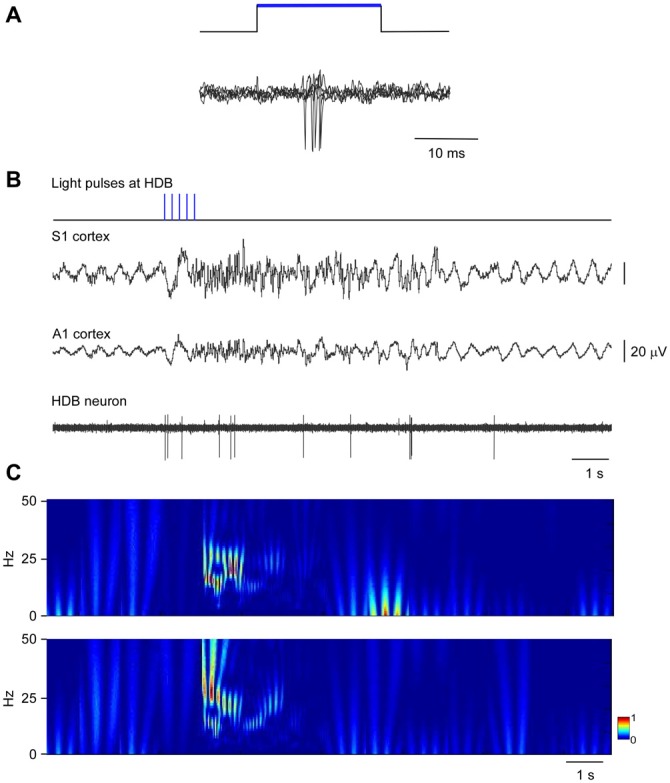
**Blue light stimulation of BF neurons induces spike firing in the BF and desynchronization of the somatosensory and auditory field potentials. (A)** A short-lasting blue LED stimuli induced spike firing in a representative B neuron (three superimposed traces are shown). **(B)** A train of stimuli (20 ms pulse duration, 5 Hz) evokes spike firing in an HDB neuron simultaneously to a desynchronization of cortical field potentials (S1 and A1 cortices). The effect lasted less than 10 s. **(C)** Wavelet analysis of the same trace shown in (**B**; S1 upper trace; A1 lower trace). Fast activity increases in both cortical field potentials after the blue light stimulation of the HDB area.

The above results indicate that neurons in HDB mainly projected to the S1 (78.2%; corresponding to 44.2% single labeled neurons and 34% double labeled neurons) whereas B neurons similarly projected to the S1 (62.6%; corresponding to 40.6% single labeled neurons and 22% double labeled neurons) and A1 (59.4%; corresponding to 37.4% single labeled neurons and 22% double labeled neurons) cortices. We used optogenetic methods for selective stimulation of cholinergic neurons in specific BF areas. To verify that blue LED stimuli induced spike firing of cholinergic neurons, we used an optrode to perform unit recordings in the BF simultaneously with optical stimulation in the same place. Short-lasting blue LED stimuli applied to the BF (HDB or B nucleus) induced spike firing in the BF neurons of ChAT-ChR2-YFP mice with a mean latency of 6.2 ± 1.1 ms (Figure 5A). All light-responsive cells (*n* = 12 cells) had slow spontaneous firing rates (0.5 ± 0.3 spikes/s). Also, a train of stimuli (20 ms pulse duration; 5 Hz) or a single pulse lasting 200 ms induced spike firing of BF neurons and a desynchronization of the cortical field potential (Figure 5B). During control conditions, the cortical field potential produced spontaneous slow oscillations reflecting a synchronized state induced by the anesthetic, which was reduced by blue light stimulation. Wavelet analysis showed that light stimulation induced an increase of fast cortical activity (>4 Hz; Figure 5C). The desynchronization in response to light stimulation lasted for only a few seconds, and could be evoked repeatedly.

**Figure 6:**
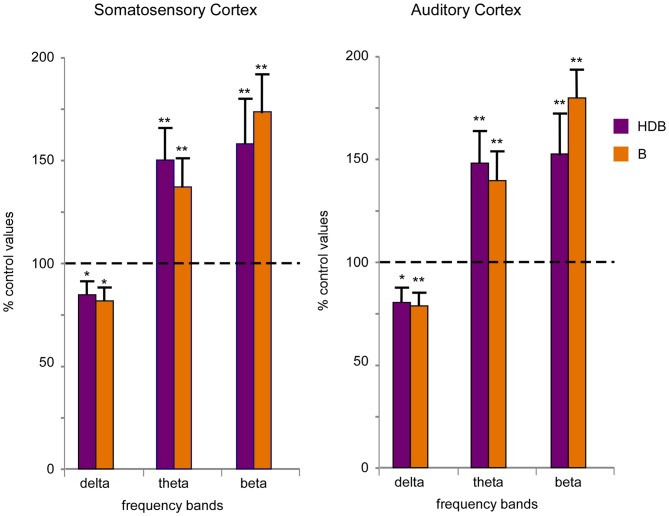
**Blue light stimulation of BF neurons induces fast activity in the cortical field potentials.** Plots of the percentage of change in the frequency band of 30 s of EEG recording respect to 30 s of a control period (100%) in the S1 and A1 cortices (left and right plots, respectively). Blue light stimulation of the HDB or B nuclei reduced the percentage of delta activity (0.5–4 Hz) and increased theta (4–10 Hz) and beta (10–30 Hz) activity during 30 s after light stimulation.

To quantify desynchronization, we compared the power spectra of the cortical field potential before (30 s; control) and after the onset of blue light stimulation (a single pulse of 200 ms duration; 30 s). Figure 6 shows the percentage change with respect to the control values (100%) in the delta frequency band (0.5–4 Hz), in the theta frequency band (4–10 Hz) and in a faster frequency band (10–30 Hz) that mainly correspond to beta frequencies. Data were calculated from six recordings in the S1 cortex and from six recordings in the A1 cortex). Blue light stimulation to the HDB reduced delta activity in the S1 and A1 cortices and increased theta frequencies and faster activities in the S1 and A1 cortices (Figure 6). The same result occurred when the blue light was applied to the B area. Although the differences were not statistically significant, HDB stimulation increased more theta frequencies in the S1 and A1 cortices than B stimulation. By contrast, B stimulation increased more beta frequencies in both cortices than HDB stimulation.

**Figure 7:**
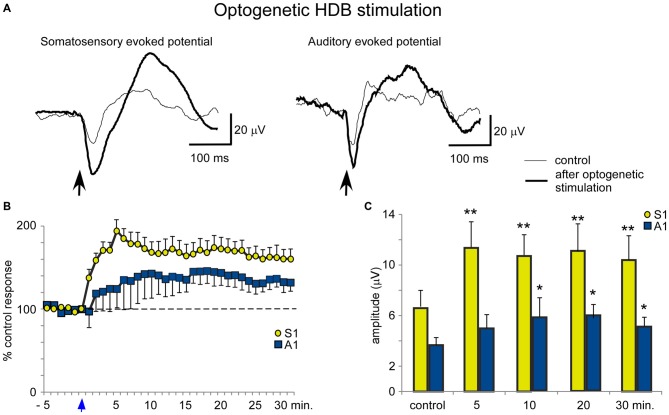
**Blue light stimulation of HDB nucleus induces an increase of somatosensory and auditory evoked potentials. (A)** Raw data shows an important increase of the somatosensory evoked potentials and a modest increase of the auditory evoked potentials (control and 5 min after blue light stimulation; thin and thick traces, respectively). **(B)** Plot shows the time course of evoked potential amplitude in 12 cases. The 100% represent the mean amplitude during the control period. Note the greater increase in somatosensory evoked potentials (S1) than in auditory evoked potentials (A1) by HDB stimulation (reference 0 s; vertical blue arrow). **(C)** Plot of the mean amplitude measured in control and at 5, 10, 20 or 30 min after blue light stimulation at HDB area. The amplitude increase was larger for somatosensory than auditory evoked potentials.

Blue light stimulation at the BF also increased the evoked potential amplitude elicited by whisker stimulation (somatosensory evoked potential) or by the application of clicks (auditory evoked potential). HDB optogenetic stimulation induced a long-lasting increase in either the somatosensory or auditory evoked potentials (Figure 7A). The effect lasted at least 30 min and was larger for the somatosensory evoked potentials than the auditory evoked potentials (Figure 7B). The mean amplitude increased rapidly from 6.6 ± 1.3 μV in the control conditions to 11.4 ± 2.0 μV, 5 min after optogenetic stimulation (ANOVA analysis, *P* = 0.002; *n* = 12) and remained 10.5 ± 1.9 μV, 30 min after stimulation (ANOVA analysis, *P* = 0.0016; *n* = 12; Figure 7C). Auditory evoked potentials were less affected by the blue light when it was directed at the HDB. The mean amplitude changed from 3.9 ± 0.6 μV in the control to 4.8 ± 1.1 μV 5 min after optogenetic stimulation (ANOVA analysis, *P* = 0.101; *n* = 12). The increase reached statistical significance 10 min after blue light stimulation (6.0 ± 1.6 μV; ANOVA analysis, *P* = 0.0109; *n* = 12) and remained facilitated 30 min after stimulation (5.2 ± 0.8 μV; ANOVA analysis, *P* = 0.0207; *n* = 12; Figure 7C).

**Figure 8:**
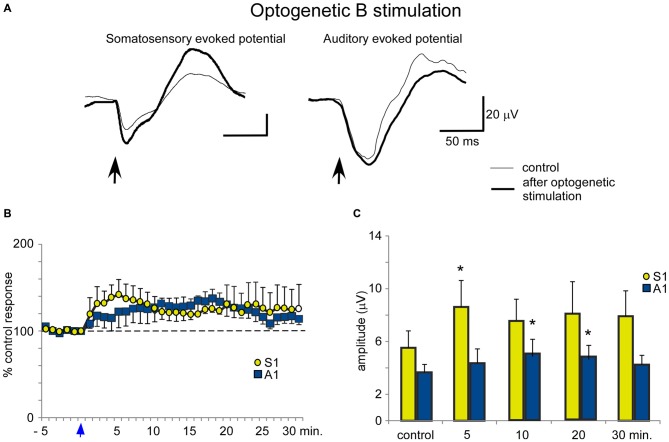
**Blue light stimulation of B nucleus induces an increase of somatosensory and auditory evoked potentials. (A)** Raw data shows that only somatosensory evoked potentials were affected by blue light stimulation at the B nucleus (control and 5 min after stimulation; thin and thick traces, respectively). **(B)** Plot shows the time course of evoked potential amplitude in 12 cases (light stimulation occurs at reference 0 s; vertical blue arrow). The 100% represent the mean amplitude during the control period. **(C)** Plot of the mean amplitude measured in control and 5, 10, 20 or 30 min after blue light stimulation at the B nucleus. The effect was smaller than after HDB stimulation.

In contrast to the HDB stimulation, B optogenetic stimulation induced a lower increase of somatosensory evoked potentials at 5 min after the application of blue light in comparison with HDB stimulation (Figures 8A,B). The mean amplitude of the somatosensory evoked potential increased from 5.5 ± 0.5 μV in the control conditions to 8.6 ± 0.9 μV, 5 min after optogenetic stimulation (ANOVA analysis, *P* = 0.0217; *n* = 12) and 7.9 ± 1.0 μV, at 30 min after stimulation (ANOVA analysis, *P* = 0.072; *n* = 12; Figure 8C). Auditory evoked potentials were also less affected by the blue light in comparison with the effect on the somatosensory evoked potentials. The mean amplitude changed from 3.7 ± 1.0 μV in the control to 4.4 ± 1.2 μV, 5 min after blue light stimulation (ANOVA analysis, *P* = 0.1338; *n* = 12), reaching statistical significance 10 min after blue light stimulation (5.2 ± 1.4 μV; ANOVA analysis, *P* = 0.0109; *n* = 12). However, the stimulation effect vanished 30 min after optogenetic stimulation (4.2 ± 1.2 μV; ANOVA analysis, *P* = 0.072; *n* = 12; Figures 8B,C).

## Discussion

A fundamental question in the present study, concerns whether the BF neuronal population operates more as a unified group, simultaneously activating all cortical areas, or as a set of distinct neuronal groups that differentially activate specific cortical regions. Our results support the latter possibility because they reveal that the BF is a heterogeneous area in which neurons projecting to different cortical areas are segregated into different neuronal groups. Most of the HDB has a large number of neurons that project to the S1 cortex (considering single and double labeled neurons), indicating that this area is specialized in sensory processing of somatosensory stimuli. By contrast, the B nucleus shows a similar number of cells projecting to the S1 and A1 cortices. Accordingly, optogenetic HDB stimulation induced more extensive facilitation of tactile evoked potentials than auditory evoked potentials. Cortical response facilitation evoked by the B nucleus stimulation was lower in both cortices and appeared more slowly in A1 than by HDB stimulation. This finding may be due to the neuronal density of the cortical projecting neurons, which is higher in the HDB area than in the B nucleus (see below).

The topography of BF projections to the cortex is an important issue because it may indicate the manner in which the cholinergic BF system participates in cortical sensory processing. The use of retrograde fluorescent neuroanatomical tracing allow us to suggest that the HDB neurons give rise to fairly widespread cortical projections but with high-density innervation focused on the S1 cortex although neurons projecting to the A1 cortex were also observed. However, we did not find a selective BF area projecting to the A1 cortex. This finding is probably due to the importance of the whisker sensory system in rodents, giving greater evidence for the existence of neuronal clusters involved in the information processing of somatosensory stimuli; this pattern of projection was also found in other sensory systems although it was less evident. In fact, the cholinergic neurons that project to V1 are located in the BF, particularly the ventral pallidum, SI and the HDB (Gaykema et al., 1990; Laplante et al., 2005). In this regard, Zaborszky et al. (2015) have demonstrated that the cholinergic and non-cholinergic pathways to the cortex are organized into segregated or overlapping pools of projection neurons. The extent of the overlap between the BF populations projecting to the cortex depends on the degree of connectivity between the cortical targets of these projection populations. By contrast, the B nucleus displayed a non-selective projection to the S1 and A1 cortices.

Previous reports have indicated regional differences in the regulation of cortical ACh release (Fournier et al., 2004; Laplante et al., 2005). This has been demonstrated in a neurophysiological experiment wherein differential modulation of somatosensory and visual cortices to tactile and visual stimuli, respectively, resulted from the activation of neighboring BF neurons (Golmayo et al., 2003). Moreover, those investigators proposed that the BF could be an anatomical and functional relay between the prefrontal cortex and sensory cortical areas. Regionally-specific activation of ACh release has also been demonstrated in visual and somatosensory cortices following the presentation of either visual or somatosensory stimuli (Fournier et al., 2004; Laplante et al., 2005). This modality-specific activation is supported by the topographical projections from the BF to sensory cortices (Zaborszky, 2002; Zaborszky et al., 2005, 2015). Taken together these results and our results suggest that cortical ACh release is increased with regional specificity in response to a specific sensory stimulus. Anatomical specificity has also been observed in the activation of prefrontal cortex-projecting vs. motor cortex-projecting BF cholinergic neurons during task performance (Parikh et al., 2007). These findings suggest the existence of different subpopulations of BF neurons involved in the modulation of specific tasks. Accordingly, clustering techniques applied to unit recordings of BF neurons during different attentional tasks have revealed a large number of distinct categories of task-phase-specific activity patterns in these BF neurons (Tingley et al., 2014). Consequently, anatomical and optogenetic results strongly suggest the existence of cholinergic neuronal populations in the BF that are involved in the modulation of sensory cortical response, as has been published recently in the somatosensory cortex (Barros-Zulaica et al., 2014; Martin-Cortecero and Núñez, 2014).

Cortically projecting neurons in the BF were characterized as cholinergic, GABAergic or peptidergic (Fisher et al., 1988; Zaborszky and Duque, 2000; Zaborszky et al., 2005; Mascagnis and McDonald, 2009). Because cholinergic neurons in the BF are scattered among neurons with different neurochemical identities, we used optogenetic stimulation of cholinergic neurons to study the specific cholinergic effects on sensory cortical responses. The optogenetic stimulation of these cholinergic HDB neurons mostly facilitated tactile evoked potentials in the S1 rather than auditory-evoked potentials in the A1 cortex. Therefore, our results support a localized cortical effect of cholinergic projections. In agreement with our data, optogenetic activation of BF cholinergic axons in the visual cortex enhances performance of a visual discrimination task, while silencing the BF cholinergic cells impaired performance (Pinto et al., 2013). However, optogenetic stimulation of cholinergic B neurons only facilitated tactile responses in the S1 while auditory-evoked potentials in the A1 cortex were less and more slowly affected. It seems that the small density of cholinergic projections from the B area to the cortex is not enough to evoke a long-lasting facilitation of sensory responses. The different result observed in the S1 and the A1 may be because the tactile stimulus was more precise (only one whisker) than the auditory stimulus (a click stimulating the entire cochlea).

In contrast to the specific long-lasting facilitation of sensory responses, blue light stimulation of either HDB or the B nucleus caused similar desynchronization of the S1 or A1 field potential during a short time period. Likewise, the illumination of neocortex desynchronizes the local field potential in the same anesthetized transgenic mice, indicating that light evoked the release of ACh in the cortex (Kalmbach et al., 2012). These findings suggest that a large network of synaptically-related cholinergic BF neurons is involved in cortical activation which is probably caused by reductions in potassium conductances (McCormick, 1992; Oldford and Castro-Alamancos, 2003). However, cholinergic modulation of precise sensory responses may be controlled by specific groups of neurons through modulation of glutamatergic cortical receptors (Carr and Surmeier, 2007; Núñez et al., 2012; Barros-Zulaica et al., 2014). In agreement with the existence of two functional roles for cholinergic BF pathways, microdialysis studies in the medial prefrontal cortex have reported a tonic ACh increase during attention-related performance tasks (Passetti et al., 2000) that may promote a general state of cortical arousal (EEG desynchronization). Moreover, ACh can also be released briefly (phasic release) in concert with cue detection in a cued appetitive response task to facilitate specific information processing (Parikh et al., 2007). Thus, phasic release of ACh would support more rapid transitions of cortical states, consistent with cholinergic regulation of attention to relevant stimuli, while a sustained ACh release could promote a general state of cortical activation (see Luchicchi et al., 2014; Sarter et al., 2014). Our results support these findings and suggest that different BF cholinergic neurons may be involved in these different roles.

## Author Contributions

MR-A and ÁN conceived and supervised all aspects of the study. IC-C and NB-Z collected all data. IC-C analyzed anatomical aspects of the data. NB-Z analyzed electrophysiological aspects of the data.

## Funding

This work was supported by a Grant from Ministerio de Economia y Competitividad (BFU2012–36107).

## Conflict of Interest Statement

The authors declare that the research was conducted in the absence of any commercial or financial relationships that could be construed as a potential conflict of interest.
